# Effect of Baking Time and Temperature on Nutrients and Phenolic Compounds Content of Fresh Sprouts Breadlike Product

**DOI:** 10.3390/foods9101447

**Published:** 2020-10-13

**Authors:** Vincenzo Alfeo, Elisabetta Bravi, Dayana Ceccaroni, Valeria Sileoni, Giuseppe Perretti, Ombretta Marconi

**Affiliations:** 1 Italian Brewing Research Centre, University of Perugia, via San Costanzo s.n.c., 06126 Perugia, Italy; vincenzo.alfeo@unipg.it (V.A.); elisabetta.bravi@unipg.it (E.B.); dayana.ceccaroni@unipg.it (D.C.); giuseppe.perretti@unipg.it (G.P.); ombretta.marconi@unipg.it (O.M.); 2 Department of Economics, Universitas Mercatorum, Piazza Mattei 10, 00186, Rome, Italy; 3 Department of Agricultural, Food and Environmental Science, University of Perugia, Borgo XX Giugno, 06121 Perugia, Italy

**Keywords:** wholegrain food, innovative product, sprouts, bread-like product, composition, antioxidants, nutritional quality

## Abstract

Sprouting has received increasing attention because of the enhanced nutritional values of the derived products. Baking affects the nutrient availability of the end products. The aim of this study was to evaluate how different baking time and temperature affect the nutritional values of bakery products derived from fresh wheat sprouts. Results indicate that the breadlike products showed comparable total polyphenol content and the thermal processes affected the free and bound fractions. Low temperature and high exposure time appear to promote the availability of the free polyphenols and sugars, while high temperature and low exposure time appear to preserve bound polyphenols and starch. Sugar profiles were influenced by baking programs with a higher simple sugar content in the samples processed at low temperature. Phenolic acids showed a strong decrease following processing, and free and bound phenolic acids were positively influenced by high baking temperatures, while an opposite trend was detected at low temperatures. Significant differences in phenolic acid profiles were also observed with a redistribution of hydroxycinnamic acids among the bound and free fractions. It may be concluded that grain type, germination conditions, and the baking programs play a fundamental role for the production of high-nutritional-value bakery products.

## 1. Introduction

In the past few years, whole grain consumption has received consumers increasing attention because of their health benefits. In parallel with the use as such, cereals are usually processed aiming to increase the amount and availability of their nutrients. The increased nutritional values of whole cereal sprouts have been widely reported by several studies and every year an increasing number of sprouted grain products are launched [1,2,3]. During sprouting the endosperm degradation occurs through specific enzymes, then the release and synthesis of the bioactive molecules takes place according to the germination extent [4,5,6]. Polyphenols represent one of the widely investigated subjects among cereal sprouts due to their antioxidant activity and are present in free, soluble conjugated and bound forms in plant material with different concentration in the grain tissues [7,8]. Phenols bound to dietary fibers, such as cell wall material, showed higher antioxidant capacity compared to the other fractions and several carbohydrate-hydrolyzing enzymes such as cellulases, amylases, hemicellulases, and glucanases, have been demonstrated to affect the release of phenolic compounds [9,10]. Furthermore, it has been reported that processing affects the release of bound phenolic compounds. Thermal treatments such as baking, roasting, boiling, and steaming appear to promote the release of bound phenolics, showing an increase of the free phenolic fraction in different food matrices [11,12]. In particular, baking, apart from the formation of reactive oxygen species, racemization of the amino acids and Maillard reactions, appears to transform bound to free phenolic acids with an apparent increase of the free fraction and a general decrease of the phenolic content probably due to the heat lability of the phenolic compounds [12,13]. Moreover, baking promotes the formation of antioxidant compounds in bread crust: for example it has been reported that ferulic acid is involved in the formation of Maillard reaction products by thermal degradation of an intermediate product such as 4-vinylguaiacol [14]. Sprouted cereal flours have been recently investigated for the inclusion in bakery products such as cakes, muffins, and cookies showing an effect on color, odor, and taste [15,16]. Despite the nutritional enhancement and the consumer acceptance due to the improvement of the sensory characteristics, the inclusion of sprouted cereal flours in bread-making processes negatively affects the rheological properties and the baking performances due to amylolytic and proteolytic activities [17,18,19]. Recent research reported that extensive germination and high inclusion (over 10%) of germinated wheat flour can result in an increase in dough stickiness and a decrease in dough strength, leading to low loaf volume, height and slice brightness of the bread [20,21]. Few previous studies have investigated the use of sprouted wheat flours for the production of a flat, unleavened bread-like products and none of these evaluated the use of fresh wheat sprouts. Moreover, to the best of our knowledge, no relevant data were found on the effect of different baking time and temperatures on the nutritional profile of bakery products. Therefore, this study aims to investigate the effect of different baking programs during the production of fresh wheat sprouts bakery products. In particular, the main quality parameters, the phenolic content, the phenolic acid and sugar profiles were evaluated for unleavened bread-like products baked using different programs and made with 100% fresh wheat sprouts.

## 2. Materials and Methods

### 2.1. Raw Materials

Common kinds of wheat (*Triticum aestivum* L.) harvested in 2018 were supplied by a local company (KeBio Srl, Gubbio, Italy). In particular, Gentil Rosso (GR) and an equal percentage mix of Verna, Inallettabile, Frassineto, Andriolo and Gentil Rosso common wheat varieties (WM), were used in this research. Cereals were germinated at 20 °C for 48 h in duplicate in an automatic malting system (Custom Laboratory Products, Milton Keynes, UK), with the aim to reach the maximum level of bioactive compounds in a short sprouting time as previously reported [6].

### 2.2. Preparation of Bakery Products

Whole fresh sprouts were ground using a laboratory meat mincer (MG51, Kenwood, Havant, UK) equipped with a 4.5 mm grinding plate. Considering the high moisture content, the resultant coarse grits were mixed as is (KMM770 Premier Mixer, Kenwood, Havant, UK) at 65 g for 10 min for the doughs formation without salt and yeast additions. The doughs, 400 g for each sample, were placed in baking pans (top length 12.5 cm, width 7.0 cm, bottom length 9.5 cm, width 5.5 cm, depth 5.0 cm, as in American Association of Cereal Chemists (AACC) method number 10-10 [22], then baked to obtain an unleavened bakery product. All the tests were conducted in duplicate.

#### Baking Programs

The baking tests were carried out in duplicate in a professional oven (Combimaster plus 061, Rational AG, Landsberg Am Lech, Germany). Two programs were designed to reach a final moisture content lower than 40% (w w^−1^) as in similar commercial products. The first baking program (150) included a temperature of 150 °C for 55 min, with 80% initial relative humidity (RH) that was lowered to 60% when the temperature probe in the product core reached 80 °C. The second baking program (100) included a temperature of 100 °C with a constant RH of 20% for 5 h. Consequently, the code GR150 and WM150 indicate the samples baked with the first baking program from the above described raw materials, while GR100 and WM100 indicate the samples baked with the second baking program.

### 2.3. Analyses

All the samples were analyzed in triplicate.

#### 2.3.1. Moisture, Nitrogen, Ash and Fat Content

Moisture, nitrogen, ash, and fat content were assessed as described in Association of Official Analytical Chemists (AOAC) methods 14.004, 945.18-B, 14.006 and American Oil Chemists Society (AOCS) 1984, Aa 4-38 respectively [23,24]. Protein content was calculated using the nitrogen conversion factor 5.7.

#### 2.3.2. Total Dietary Fiber

Total dietary fiber (TDF) was determined by means of Megazyme assay kit (Megazyme International, Ireland) following the AOAC method 985.29 [23].

#### 2.3.3. Carbohydrates

Carbohydrates were calculated as the percentage difference with moisture, proteins, ashes, fats and TDF.

#### 2.3.4. Water Activity

The water activity (a_w_) was determined using an Aqualab^®®^ series 3 (Decagon, Pullman, Washington, DC, USA) calibrated with potassium chloride solution (a_w_ = 0.984 ± 0.003).

#### 2.3.5. Extraction of Free and Bound Fractions of Phenols and Phenolic Acids

Extraction of free and bound fractions of phenols and phenolic acids was carried out following the method proposed by Stagnari et al., 2017 [25]. Samples (1 g) were added to a 15 mL extraction solution of methanol, water, acetic acid (70:29.5:0.5), homogenized for 1 min on ice using an ultraturrax (8000 rpm), ultrasonicated for 40 min on ice and centrifuged for 10 min at 4235× *g*. The extraction was carried out twice and the supernatants were vacuum dried and suspended in the HPLC analysis injection eluent A (0.1 M citric acid and 0.2 M sodium hydrogen phosphate; 85:15; v v^−1^). The remaining pellet was used for the extraction of the bound fraction. Alkaline hydrolysis (4 M sodium hydroxide) at room temperature was used to release the bound fraction from the pellet, then the pH was adjusted to 2 and phenols and phenolic acids were extracted three times with 20 mL of ethyl acetate, as reported above for the free fraction on ice by utraturrax (8000 rpm), then centrifuged for 10 min at 4235× *g*. The supernatants were vacuum dried and suspended as reported above for the free fractions.

#### 2.3.6. Determination of Phenol Content

The Folin–Ciocalteu method was used to assess the total polyphenol content of free and bound fractions [26]. The absorbance was measured at 760 nm using gallic acid as a standard and results were expressed as mg of the gallic acid equivalent (GAE) g^−1^ of the sample. Total polyphenol levels were calculated as the sum of the free and bound fractions.

#### 2.3.7. Determination of Phenolic Acids by High Pressure Liquid Chromatography (HPLC) System

Azura MWD 2.1 L UV–Vis detector (Knauer, Berlin, Germany) coupled with a quaternary Azura P 6.1 L pump was used to detect free (FPA) and bound (BPA) phenolic acids. Samples were injected using a Knauer 3950 autosampler with a 10 μL loop. A SunShell C18 column (ChromaNik Techmologies Inc. 50 mm × 2.1 mm ID) was used for the phenolic acids separation at room temperature with a flow rate of 0.4 mL/min. Mobile phase A was 0.1 M citric acid and 0.2 M sodium hydrogen phosphate (85:15; v v^−1^). Mobile phase B was prepared with phase A, methanol, acetonitrile (30:20:50). 85% orthophosphoric acid was used to adjust the pH mobile phases at 3.44. The elution gradient was obtained using mobile phase A 90% (0 min), 100% (2 min), 70% (8 min), 50% (10 min), 20% (12 min), 90% (12.5 min) and the peaks were detected at 254, 278, and 324 nm. Data acquisition was completed with Clarity Chromatography Software (DataApex, Prague, Czech Republic). Calibration curves were obtained through standard compounds and the linearity of calibration plots was between 0.3 and 6 μg mL^−1^.

#### 2.3.8. Determination of Free Sugars Profile

Free sugar extraction was carried out with 10 g of sample, homogenized in 100 mL 80% ethanol solution using an ultraturrax, heated 5 min in a boiling water bath, then cooled at room temperature and centrifuged at 4235× *g* for 10 min. Supernatant was vacuum evaporated and resuspended to a known volume in distilled water. Sugar profile was obtained by HPLC coupled with an evaporative light scattering detector (ELSD) following the method proposed by Floridi, Miniati, Montanari, and Fantozzi, (2001) [27].

#### 2.3.9. Statistical Analysis

Analysis of variance (one-way ANOVA applying Tukey’s post hoc test) and principal component analysis (PCA) were performed using Matlab R2015a (MathWorks, Inc., Nutick, MA, USA).

## 3. Results and Discussion

**Figure 1:**
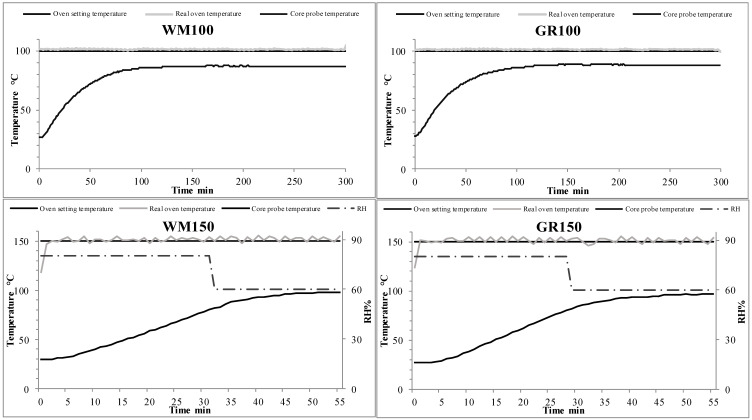
Oven parameters registered during baking tests. GR = Gentil Rosso; WM = wheat mix; 100 = baking temperature 1; 150 = baking temperature 2; RH = relative humidity.

Figure 1 shows the oven setting parameters and the trend of the registered temperatures of the baking tests, the core temperature of the samples as well as the RH. The baking programs were carried out using different temperatures, 100 and 150 °C, for 300 and 55 min respectively. The real oven temperatures were closer to the setting point and the probe shows that the samples’ cores reached the enzymatic degradation temperature of 80 °C in 67 and 64 min for WM and GR respectively when baked at 100 °C, while, at 150 °C, this took 31 and 28 min. For the tests carried out at 100 °C, for both samples, the core temperature probe never exceeded 90 °C during the baking time, while, for those carried out at 150 °C, the core probe never detected a temperature higher than 100 °C. The RH was constant at 20% when the samples were baked at 100 °C and modulated as shown in Figure 1 for the tests at 150 °C to avoid crust formation on the sample upper surface at the beginning of the process that can affect the moisture reduction during baking. The obtained samples show similar visual aspects, as displayed in supplementary material (Figure S1).

The main quality attributes of the different bakery products are summarized in Table 1.

As expected, the moisture content was lower than 40% and comparable for all the samples, no significant differences were observed for GR samples, while, for WM100, the moisture was slightly higher than WM150. The a_w_ ranged between 0.96 and 0.97 and no significant differences were observed between samples baked at different temperatures. These results were similar to moisture and a_w_ showed in other research concerning bread-baking tests [28,29]. With regard to proteins, no significant differences were observed between the GR baking tests, while, for WM, a slightly higher content was detected for the test at 150 °C. The ash ranged between 1.2 and 1.4%, respectively for WM and GR, while fat content was comparable in WM samples and slightly higher in GR100. The TDF was higher in WM samples and no significant differences were observed for the tests carried out with the different baking programs. The amount of carbohydrates was about 43.7% for GR samples, while, in WM samples, it was about 42.0%. The level of proteins, ash, fats, TDF and carbohydrates were in line with those reported for different bread and breadlike products [30,31,32]. The amount of carbohydrates converted into simple sugars during sprouting and baking are reported in Table 1. It is interesting to note that samples baked at 100 °C show significantly higher sugar content compared to those processed at 150 °C. The sugars accounted for 12.5 and 13.9% of the total carbohydrates detected respectively for GR100 and WM100, while, for GR150 and WM150, the sugars accounted for 9.9 and 10.4% respectively. The higher sugar content observed for the samples baked at 100 °C was probably due to the enzymes that were active for a longer time during the tests and the polysaccharides were hydrolyzed into sugars and dextrins.

The sugar profile of the bakery products is shown in Table 2, and an example of the chromatogram is provided as supplementary material (Figure S2).

Fructose levels were comparable for all the samples, glucose significantly differed between samples and the higher level was observed in samples baked at the lower temperature. Sucrose levels were comparable among GR samples, while, for WM, a higher content was detected in the test carried out at 150 °C. Maltose was the most represented sugar in all the samples and a significantly higher content was detected in the samples baked at 100 °C. These results were in line with the literature for sprouted wheat flours and 100% sprouted wheat bread [17,32]. The sugar profile appears to be influenced by the baking program. For the samples baked at 100 °C, the sum of fructose, glucose, sucrose and maltose accounted for more than half of the total sugars, namely 52.7 and 51.9% respectively for GR100 and WM100. The samples baked at 150 °C showed an opposite trend and dextrins accounted for 54.1 and 54.4% of the total sugar content respectively for GR and WM. With regard to the dextrins pattern, no significant differences were observed among the different baking tests; maltoheptaose was the most represented dextrin for GR150, WM100 and WM150, while it was maltoexahose for GR100. Total sugar content was in the range 15.48–16.94 g 100 g^−1^ dry basis (db) for GR samples and 15.71–18.82 g 100 g^−1^ (db) for WM samples respectively for the baking tests carried out at 150 and 100 °C. The different baking tests appear to affect the sugar content and profile with significant differences among the samples. In particular, the total sugar content of the GR and WM raw sprouts was respectively 1.41 and 1.62 g 100 g^−1^ (db) and no detectable levels of dextrins were observed in the same samples previously studied [6]. The starch hydrolysis which occurs during early baking led to an increase in dextrins, as a result of the amylase activities at a temperature lower than 80 °C, as reported in other research [33]. As mentioned above, after processing, the main sugar was maltose and sucrose levels were lower compared to the raw sprouts. The reduction in sucrose was 17.3 and 4.9% for GR100 and GR150, while WM100 and WM150 accounted for 60.0 and 27.0% of the levels detected in raw sprouts. This was probably due to the thermal and enzymatic sucrose degradation occurred during sprouting and baking [34].

**Figure 2:**
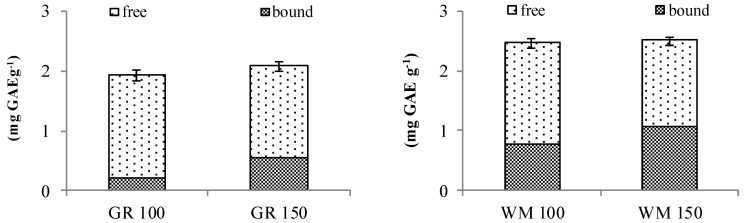
Free and bound polyphenols content of the sprouted grains bakery products (mg GAE g^−1^ db) *; * n = 3 replicates; GR = “Gentil Rosso”; WM = “wheat mix”; 100 = baking temperature 1; 150 = baking temperature 2.

Figure 2 shows the free, bound and total polyphenols content of the bakery products. No significant differences were observed for the polyphenols content among the tests carried out at different temperatures and the level ranged between 1.92–2.07 mgGAE g^−1^ (db) for GR samples and 2.47–2.51 mgGAE g^−1^ (db) for WM, respectively, for the samples baked at 100 and at 150 °C. Furthermore, the total polyphenol content measured in GR and WM bakery products was similar to the level detected in the raw sprouts, respectively 2.11 and 2.45 mgGAE g^−1^ (db) [6]. In our tests, the total polyphenol content would not appear to be influenced by the baking process and these results were in contrast with another research highlighting a reduction of the total phenol content following bread making with sprouted flours [19]. Free polyphenols represent the main fraction of the polyphenols in our samples and differences were observed between the baking tests. GR100 showed a free polyphenol content of 1.74 mg GAE g^−1^ (db) which was significantly higher than 1.55 mgGAE g^−1^ (db) assessed in GR150. In WM samples the free polyphenols fraction was comprised between 1.73 and 1.46 mg GAE g^−1^ (db) respectively for the samples baked at 100 and 150 °C. Bound polyphenols differed among samples, GR100 showed a content of 0.18 mgGAE g^−1^ (db) which was significantly lower when compared to 0.52 mg GAE g^−1^ (db) detected in GR150. The same trend was observed for WM samples, the bound polyphenols were in the range 0.74–1.05 mgGAE g^−1^ (db) respectively for WM100 and WM150. Although total polyphenol content was comparable between raw sprouts and bakery products, the thermal processes affected the amount of the free and bound fractions to a different extent. The baking process appeared to increase the availability of the free fraction through the release of the bound polyphenols as reported in the literature [7]. In particular, for GR100 and GR150, the increase of the free polyphenol fraction accounted for 24.3 and 10.7%, while the bound fraction decreased respectively by 74.6 and 26.8% when compared to GR raw sprouts. In WM100 and WM150, the increase of the free fraction was 32.1 and 11.5%, and, for the bound polyphenols, the reduction accounted for 35.1 and 7.9% when compared to WM raw sprouts. These results show that longer baking time appears to increase the free polyphenol fractions. This could reflect on antioxidant activity; in fact, according to relevant literature, the antioxidant capacity is positively affected by increasing baking time, temperature and sugar amount, but the main effect was due to the baking time [35]. Furthermore, the release of polyphenols from the food matrices increases their bioavailability and makes available these nutraceutical compounds for the intestinal absorbtion [36].

Phenolic acids (PA) in cereals are the main antioxidant contributors and about 75% are present in bound form [12,13]. The PA detected in our samples are shown in Table 3.

Sinapic and ferulic acids were the most represented free PA (FPA) for all the samples, no significant differences were observed in sinapic level between the 100 and 150 °C baking tests, while for ferulic acid, the WM100 sample showed a higher content. GR samples showed a comparable level of total FPA with a slightly higher content in GR150 while WM150 showed a significantly higher level when compared to WM100. The GR and WM raw sprouts showed differences in content and profile of the FPA when compared to the bakery products. In particular, for GR raw sprouts, FPA accounted for 127.15 μg g^−1^ mainly consisting of vanillic acid (117.07 μg g^−1^) [6] that was almost totally degraded during both baking tests, and the reduction of the total FPA content was 43.64 and 47.24% respectively for GR150 and GR100. The same trend was observed in WM, the total FPA level in raw sprouts was 59.15 μg g^−1^ mainly consisting of vanillic acid that was almost completely degraded during baking. The extent of the total FPA reduction in WM samples was 37.09 and 10.09% respectively for WM100 and WM150. The reduction of the FPA following baking tests has been reported in several studies [37,38]. With regard to bound PA (BPA), the most represented were ferulic and sinapic acids for both WM samples and for GR150, while, in GR100, salicylic and ferulic acids were dominant. The total BPA levels showed significant differences with a higher content for the samples baked at 150 °C, but, when compared to the raw sprouts, a significant decrease was detected in all samples. In particular, the total BPA reduction was 95.24 and 85.59% for GR100 and GR150, and 83.80 and 79.77% respectively for WM100 and WM150, mainly due to the release and partial degradation of the hydroxycinnamic acids such as ferulic and sinapic during baking tests. An opposite trend was observed for the hydroxybenzoic acids. The bound salicylic acid was higher in samples baked at lower temperature and was not found in WM150, which showed the higher level of free syringic acid. In our tests the bound hydroxybenzoic acids were released and partially degraded with higher intensity during the test carried out at higher temperature and lower exposure time. These results, even if to a higher extent, were in line with those reported in other research showing a strong reduction of BPA following baking and other food processes [7,13]. In fact, heat stress could cause degradation of free and soluble conjugated PA and the release and partial degradation of the bound PA [39]. Considering that the most part of the PA were present in bound form, the total PA (TPA) content followed the BPA trend. GR150 and WM150 were the samples with a significantly higher content when compared to those baked at 100 °C. Indeed, the extent of the total PA reduction was 88.56 and 79.75% in GR samples, and 80.68 and 75.11% in WM samples, respectively for the tests carried out at 100 and 150 °C. The impact of the food processing on PA content and stability depends on the treatment intensity and time as well as food matrices [11].

**Figure 3:**
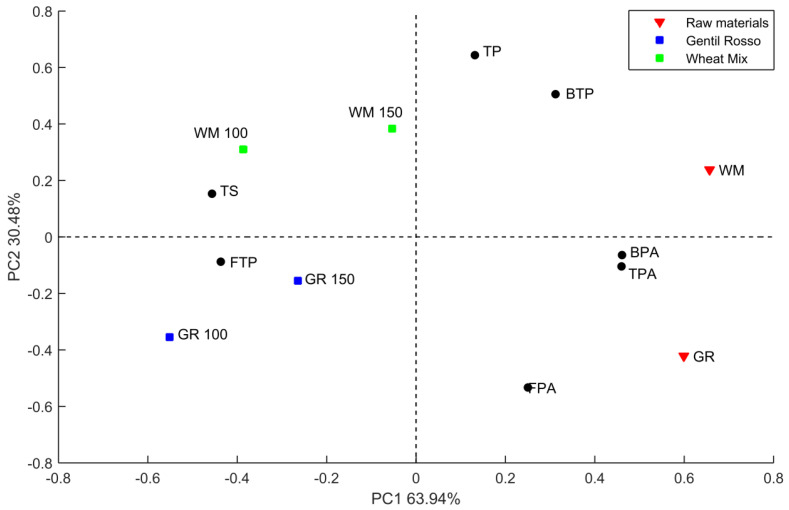
Biplot of the scores and loading of the PCA. GR = Gentil Rosso; WM = wheat mix; 100 = baking temperature 1; 150 = baking temperature; FTP = free total polyphenols; BTP = bound total polyphenols; TP = total polyphenols; FPA = free phenolic acids; BPA = bound total polyphenols; TPA = total phenolic acids; TS = total sugars.

Figure 3 shows the biplot of scores and loading of the principal component analysis (PCA) carried out to discriminate samples according to their main characteristics. The variables selected for the PCA were the free, bound and total polyphenols, the free, bound and total phenolic acids, and the total sugar content (including dextrins). The selected principal components (PC) can explain over 94% of the variances, respectively 63.94% from the PC1 and 30.48% from the PC2. The PC1 spread the samples scores according to the intensity of the phenolic release and starch degradation during the processes. In fact, the raw material sample scores were plotted on the positive side of the PC1, where the greater influence was due to the level of BPA and TPA. From the positive to the negative side of the PC1, we found the scores of the samples baked at 150 °C, then the scores of the samples baked at 100 °C. These samples were characterized by increasing levels of sugar and free polyphenols, indicating that the lower temperature and the high exposure time promoted the starch degradation and the release of the bound polyphenols.

The PC2 discriminated samples according to genotypes characteristics, from the positive side according to the presence of total and bound polyphenols, while from the negative side for the level of FPA. Samples scores plotted on the positive side of the PC2 were WM samples that were characterized by the higher level of total and bound polyphenols, while on the negative side we found the GR150 closer to the positive side and then GR 100 with higher influence of FPA when compared to WM. The relative position of the scores of the samples baked at 150 °C compared respectively to the same samples baked at 100 °C was due to the effect of the thermal treatments on the bound polyphenols that discriminate the samples according to PC2. This could indicate that, in our tests, higher temperature and low exposure time reduced the release of bound polyphenols.

## 4. Conclusions

One hundred percent sprouted wheat bakery products can represent a valuable source of compounds with high nutritional value. The raw sprouts, as well as the baking conditions, such as temperature and time, affect the release of the phenolic compounds and starch degradation into simple sugars and dextrins. The baking program characterized by high temperature and short exposure time appears to impact less on bound polyphenols and sugar release when compared to the other baking program. When samples were baked at low temperature for a long exposure time the results show a higher intensity in phenolic and sugar release. Although the level of the total polyphenols remained substantially unchanged during the process, the PA content and profiles appear to be affected by the baking programs. Our tests showed a strong reduction of the phenolic acid following baking, with a greater impact of the baking program carried out at 100 °C. The PA profiles show a reduction of BPA, such as ferulic and sinapic, and an increase of the free form of the same hydroxycinnamic acids. Furthermore the FPA found in raw sprouts were almost totally degraded during baking and were substituted by the BFA released during baking to a different extent depending on the baking time and temperature. It may be concluded that grain type, germination conditions, and the baking program play a fundamental role for the production of high-nutritional value bakery products.

## Figures and Tables

**Table 1:** Bakery products (breadlike) quality attributes *.

	GR 100	GR 150	WM 100	WM 150
Moisture%	38.71 ± 0.31 ^a^	38.59 ± 0.34 ^a^	39.40 ± 0.17 ^B^	38.66 ± 0.01 ^A^
a_w_	0.96 ± 0.01 ^a^	0.97 ± 0.01 ^a^	0.96 ± 0.01 ^A^	0.96 ± 0.01 ^A^
Proteins %	7.83 ± 0.01 ^a^	7.84 ± 0.01 ^a^	7.88 ± 0.01 ^A^	8.01 ± 0.01 ^B^
Ash %	1.40 ± 0.10 ^a^	1.44 ± 0.10 ^a^	1.21 ± 0.09 ^A^	1.21 ± 0.09 ^A^
Fat %	0.50 ± 0.01 ^b^	0.42 ± 0.01 ^a^	0.47 ± 0.01 ^A^	0.48 ± 0.01 ^A^
TDF %	7.80 ± 0.55 ^a^	7.93 ± 0.51 ^a^	8.54 ± 1.00 ^A^	9.55 ± 0.97 ^A^
Carbohydrates %	43.76 ± 0.76 ^a^	43.78 ± 0.94 ^a^	42.50 ± 0.94 ^A^	42.09 ± 1.07 ^A^
- of which sugars *^+^* (g 100 g^−1^)	5.55 ± 0.59 ^b^	4.37 ± 0.07 ^a^	5.94 ± 0.13 ^B^	4.40 ± 0.37 ^A^

^+^ (Sum of fructose, glucose, sucrose and maltose); * n = 3 replicates; GR = Gentil Rosso; WM = wheat mix; 100 = baking temperature 1; 150 = baking temperature 2; a_w_ = water activity TDF = total dietary fiber. For each class of samples, values in the same row followed by different letters (lower case for GR and upper case for WM) are statistically different (*p* < 0.05).

**Table 2:** Sugar profile of the bakery products (g 100^−1^ db) *.

Sugar	GR 100	GR 150	WM 100	WM 150
Fructose	0.54 ± 0.13 ^a^	0.41 ± 0.02 ^a^	0.41 ± 0.02 ^A^	0.49 ± 0.12 ^A^
Glucose	1.94 ± 0.25 ^b^	1.14 ± 0.12 ^a^	2.03 ± 0.21 ^B^	1.15 ± 0.07 ^A^
Sucrose	0.67 ± 0.12 ^a^	0.77 ± 0.11 ^a^	0.40 ± 0.01 ^A^	0.73 ± 0.16 ^B^
Maltose	5.91 ± 0.06 ^b^	4.79 ± 0.35 ^a^	6.90 ± 0.76 ^B^	4.80 ± 0.22 ^A^
D3 (maltotriose)	2.22 ± 0.01 ^a^	1.96 ± 0.34 ^a^	1.94 ± 0.30 ^A^	1.57 ± 0.21 ^A^
D4 (maltotetraose)	1.47 ± 0.43 ^a^	0.90 ± 0.14 ^a^	1.53 ± 0.23 ^A^	1.16 ± 0.16 ^A^
D5 (maltopentaose)	0.74 ± 0.22 ^a^	0.83 ± 0.24 ^a^	0.91 ± 0.03 ^A^	0.91 ± 0.06 ^A^
D6 (maltohexaose)	2.19 ± 0.21 ^a^	1.90 ± 0.14 ^a^	1.75 ± 0.35 ^A^	2.20 ± 0.54 ^A^
D7 (maltoeptaose)	1.41 ± 0.30 ^a^	2.79 ± 0.87 ^a^	2.94 ± 0.19 ^A^	2.68 ± 0.32 ^A^
Total	17.09 ± 1.24 ^b^	15.48 ± 0.08 ^a^	18.82 ± 0.67 ^B^	15.71 ± 0.68 ^A^

db = Dry basis; * n = 3 replicates; GR = Gentil Rosso; WM = wheat mix; 100 = baking temperature 1; 150 = baking temperature 2. For each class of samples, values in the same row followed by different letters (lower case for GR and upper case for WM) are statistically different (*p* < 0.05).

**Table 3:** Phenolic acid (PA) profile of the bakery products (µg g^−1^ db) *.

	GR 100	GR 150	WM 100	WM 150
***FPA***				
vanillic	4.79 ± 0.84 ^a^	5.30 ± 0.13 ^a^	-	3.77 ± 0.17
syringic	3.21 ± 0.93	-	-	9.64 ± 0.39
salicylic	-	-	4.71 ± 0.30	-
caffeic	2.71 ± 0.33 ^a^	4.01 ± 0.42 ^b^	2.58 ± 0.04 ^A^	2.63 ± 0.06 ^A^
ferulic	14.07 ± 0.42 ^a^	12.37 ± 1.36 ^a^	13.03 ± 0.59 ^B^	9.80 ± 0.65 ^A^
m-coumaric	11.08 ± 2.01 ^a^	12.11 ± 0.03 ^a^	-	-
sinapic	31.22 ± 1.42 ^a^	37.86 ± 4.77 ^a^	16.89 ± 1.56 ^A^	19.38 ± 1.16 ^A^
*total FPA*	*67.08 ± 5.95 ^a^*	*71.65 ± 6.70 ^a^*	*37.21 ± 2.48 ^A^*	*53.18 ± 3.86 ^B^*
***BPA***				
syringic	2.06 ± 0.36 ^a^	2.59 ± 0.07 ^a^	3.49 ± 0.44	-
salicylic	28.37 ± 5.21 ^b^	5.96 ± 0.38 ^a^	8.03 ± 1.08	-
ferulic	5.52 ± 0.89 ^a^	82.93 ± 6.92 ^b^	97.45 ± 6.99 ^A^	133.13 ± 6.28 ^B^
sinapic	1.40 ± 0.33 ^a^	21.74 ± 2.86 ^b^	24.87 ± 0.31 ^A^	25.12 ± 4.04 ^A^
caffeic	-	-	-	4.82 ± 1.11
p-coumaric	-	-	-	4.07 ± 0.83
*total BPA*	*37.36 ± 5.02 ^a^*	*113.22 ± 9.47 ^b^*	*133.84 ± 8.82 ^A^*	*167.14 ± 12.25 ^B^*
Total PA	104.45 ± 8.26 ^a^	184.86 ± 15.85 ^b^	171.05 ± 11.30 ^A^	220.33 ± 16.11 ^B^

db = Dry basis; * n = 3 replicates; GR = Gentil Rosso; WM = wheat mix; 100 = baking temperature 1; 150 = baking temperature 2; FPA = free phenolic acids; BPA = bound phenolic acids. For each class of samples, values in the same row followed by different letters (lower case for GR and upper case for WM) are statistically different (*p* < 0.05).

## Supplementary Materials

The following are available online at https://www.mdpi.com/2304-8158/9/10/1447/s1, Figure S1: Bread-like products, Figure S2: Sugar profile chromatogram of the GR100 sample.
