# Supplementary figures and images for: Effect of Baking Time and Temperature on Nutrients and Phenolic Compounds Content of Fresh Sprouts Breadlike Product
Source: Foods. 2020 Oct 13;9(10):1447. doi: 10.3390/foods9101447 (PMC7599486; doi:10.3390/foods9101447)

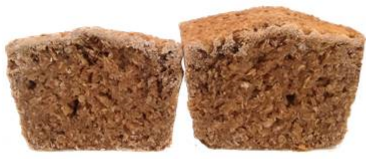

GR100

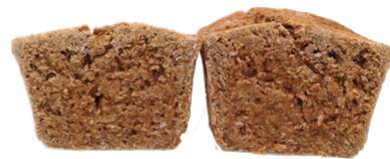

GR150

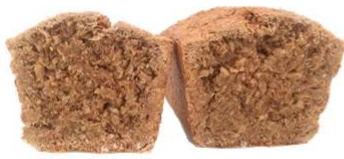

WM100

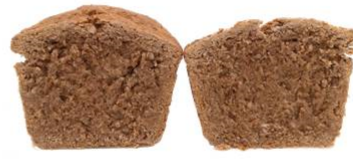

WM150

**Figure 1s.** Bread-like products

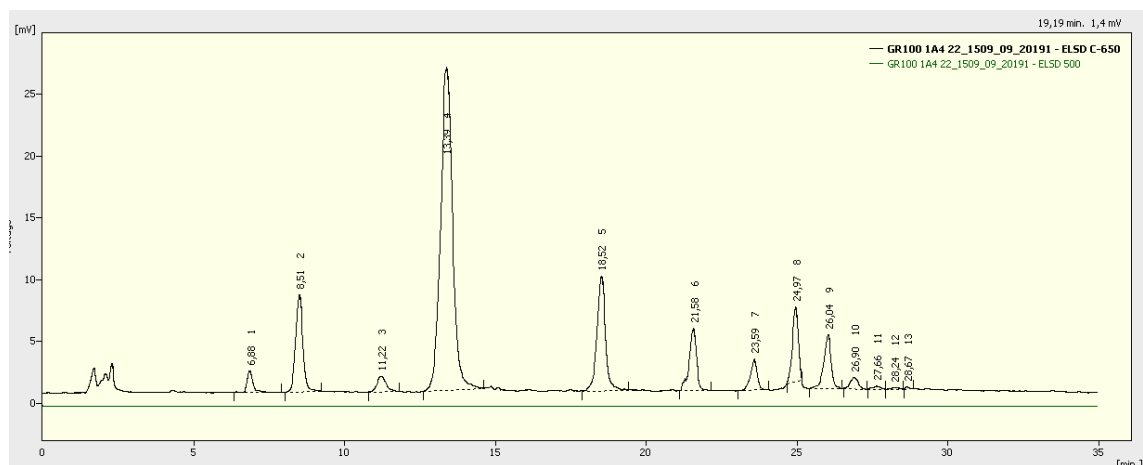

**Figure 2s.** Sugar profile chromatogram of the GR100 sample.

Supplement: Supplementary file 1 [file foods-09-01447-s001.pdf]
